# An ultra-compact leaky-integrate-and-fire model for building spiking neural networks

**DOI:** 10.1038/s41598-019-47348-5

**Published:** 2019-07-31

**Authors:** M. J. Rozenberg, O. Schneegans, P. Stoliar

**Affiliations:** 1 Laboratoire de Physique des Solides, UMR8502 CNRS - Université Paris-Sud, Université Paris-Saclay, 91405 Orsay, Cedex France; 2 Laboratoire Génie électrique et électronique de Paris, CentraleSupélec, UMR8507 CNRS – Sorbonne Université, Université Paris-Saclay, 91192 Gif-sur-Yvette, Cedex France; 3 National Institute of Advanced Industrial Science and Technology (AIST), 305-8565 Tsukuba, Japan

**Keywords:** Electronic properties and materials, Applied physics, Electrical and electronic engineering, Electronics, photonics and device physics

## Abstract

We introduce an ultra-compact electronic circuit that realizes the leaky-integrate-and-fire model of artificial neurons. Our circuit has only three active devices, two transistors and a silicon controlled rectifier (SCR). We demonstrate the implementation of biologically realistic features, such as spike-frequency adaptation, a refractory period and voltage modulation of spiking rate. All characteristic times can be controlled by the resistive parameters of the circuit. We built the circuit with out-of-the-shelf components and demonstrate that our ultra-compact neuron is a modular block that can be associated to build multi-layer deep neural networks. We also argue that our circuit has low power requirements, as it is normally off except during spike generation. Finally, we discuss the ultimate ultra-compact limit, which may be achieved by further replacing the SCR circuit with Mott materials.

## Introduction

We are currently witnessing an ongoing technological revolution. The longstanding promise of artificial intelligent systems realized in neural networks is beginning to materialize^1^. Significant milestones have been overcome such as, for instance, the deep neural network algorithm AlphaGo beating the world champion of the board game go. A neural network is a system of interconnected units, which is inspired by the mammalian brain. The units, called neurons, perform a simple basic non-linear process, and their inter-connections are called synapses^2^. Neural network systems are implemented by either running software on a conventional (super) computer, as AlphaGo^3^, or directly in hardware by dedicated integrated CMOS (VLSI) circuits^4^. A notable example of the latter is the chip TrueNorth, whose circuits emulate both, synaptic and neuronal functionalities^5^. However, both strategies suffer from significant bottlenecks to achieve the massive scale needed to compete with a mammalian brain. It is often quoted the amazing power efficiency of the human brain, which counts 10^11^ neurons and 10^15^ synapses and requires just about 20 W to function. In contrast, running AlphaGo on a digital supercomputer requires the order of hundreds of kWs. Nevertheless, conventional electronics is not to be blamed for lack of efficiency, as the last generation of microprocessors in modern digital computers and smartphones can integrate 10^10^ transistors and consume less than 10 W. Moreover, the brain-inspired chip TrueNorth counts 5.4 × 10^9^ transistors and consumes less than 0.07 W^5^. While this is impressive, the implementation of a circuit that emulates *neuronal* function currently requires a large number of transistors. In TrueNorth, each of its 4096 cores has 1.2 million transistors that implement 256 neurons. Hence, a neuron requires about 10^4^ transistors. This indicates that there is a need to explore ways of building efficient neuromorphic circuits with a significant reduction in the number of components. Such *compact* neuron models have been proposed, which typically require tens of transistors^6,7^. Here, we present a significant improvement along this direction and introduce an *ultra-compact* neuron model that brings the count of active devices down to three, two transistors and a silicon controlled rectifier (SCR), also called thyristor. We identify the non-linear I-V characteristics and the gate of the SCR as the key features which enable a simple implementation of an electronic neuron with the *leaky-integrate-and-fire* (LIF) model functionality^8^.

## The Ultra-Compact Leaky-Integrate-and-Fire Neuron Model

**Figure 1:**
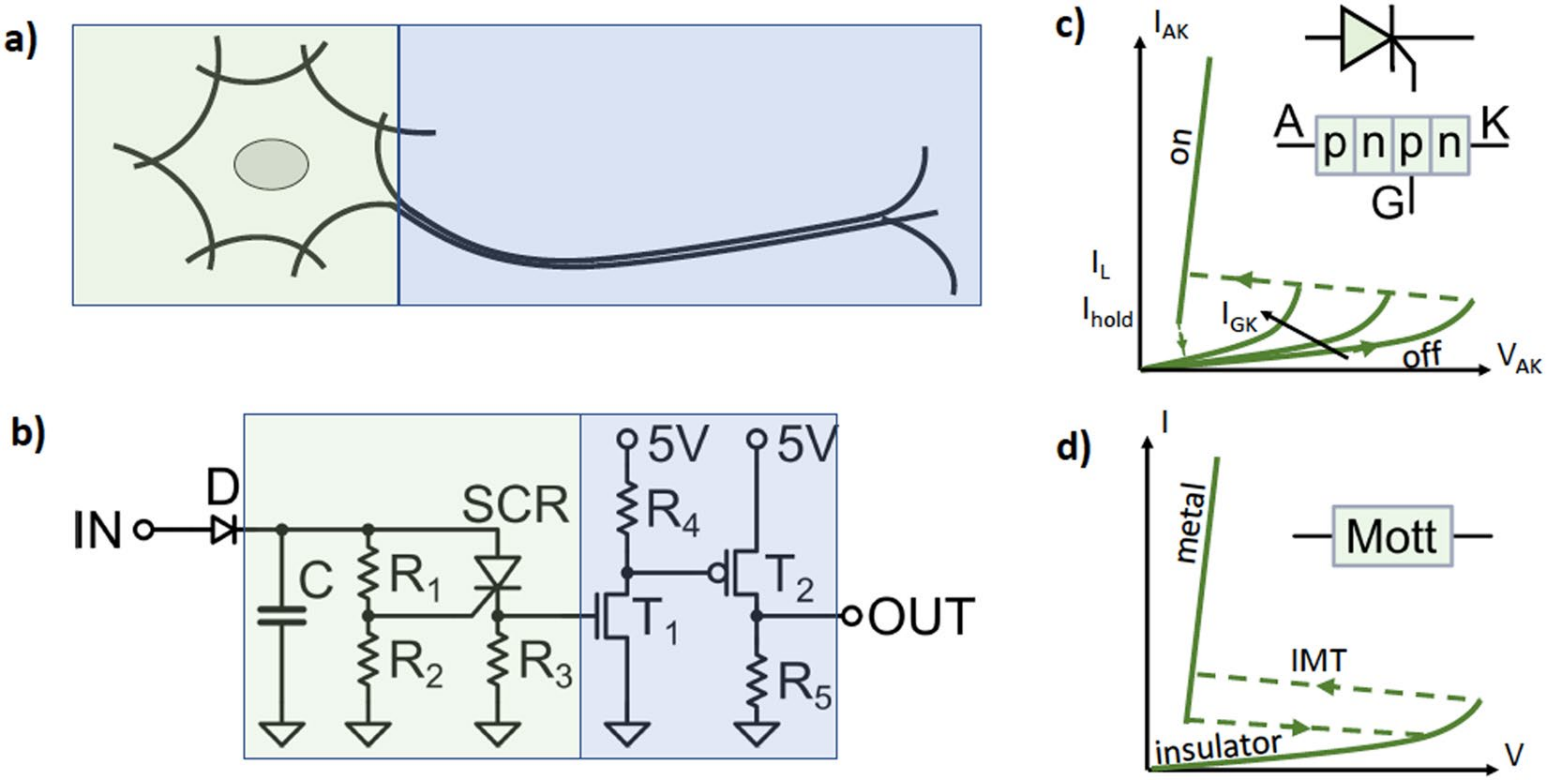
Panel (a) shows a schematic view of a biological neuron where two regions are indicated by the color boxes: integration and spike generation soma (green) and spike propagating axon (blue). Panel (b) shows the electronic circuit of the UC neuron that we call type I. The colored regions indicate the parts of the circuit that implement the analogue functionalities. The diode D is not considered part of the UC neuron but of the input circuit. Panel (c) shows the schematic I-V characteristics of a SCR device. A, K and G stand for anode, cathode and gate. I_L_ and I_hold_ are the “latch” and “hold” currents. The off-on transition is controlled by the gate. Panel (d) depicts the schematic I-V characteristics of a Mott insulator. The insulator-metal transition may be controlled by Joule heating or applied electric field^2^.

The circuit of our ultra-compact (UC) neuron is shown in Fig. 1, where we draw a qualitative analogy with a schematic biological neuron. This LIF neuron exploits the I-V characteristic of a conventional electronic component, namely, the SCR. This device is realized by a four layer *pnpn* structure, which may be integrated into standard micro-electronics^9^. The key feature of the SCR is that is has a diode-like behavior with threshold and hysteresis that can be controlled by a gate.

The *leaky* and *integrate* features are naturally implemented by a RC pair. The capacitor (C) *integrates* the charge of incoming current spikes, which may *leak* out through the resistor (R = R_1_ + R_2_) during the time intervals between spikes. The key *fire* feature of our model is realized by the SCR’s voltage threshold, which is set by its anode-cathode tension and is tuned by the gate, through the resistors R_1_ and R_2_. When the voltage threshold is attained, the SCR switches to the on-state and the capacitor quickly discharges through the small R_3_, generating a spike of current. The SCR remains in the on-state until the currents decreases to the value *I*_*hold*_, when the capacitor is almost fully discharged. This process can be associated to the relaxation or refractory period of the artificial neuron. In order for the spike to be able to drive a downstream neuron, the strength of the signal needs to be reinforced. As shown in Fig. 1, this is implemented by a pair of MOS transistors that play the role of the axon. Thus, our UC neuron is implemented with just one SCR and two transistors, plus one “membrane” capacitor and a few resistors. This solution, by construction, has likely a minimal number of components. In fact, we have identified each of the three features of the *leaky*-*integrate*-and-*fire* model with three respective devices, a *resistor*, a *capacitor* and a *SCR*. These components realize the non-linear process of threshold spike generation in the “soma” of the artificial neuron.

We should also mention that the I-V characteristic of the SCR bears a strong similarity with that of the Mott materials, which we schematically depict in Fig. 1. In fact, Mott materials are been intensively investigated for neuromorphic electronic devices, including artificial neurons^2^. The key feature of those systems is that they present an insulator to metal first-order phase transition, which may be driven by temperature or applied electric field.

## Results

In the following, we demonstrate the behavior of our LIF neuron model. We have implemented the electronic circuit with out-of-the-shelf components (see Table 1 in *Methods* below) and obtained readings of several input and output voltages. We also monitored the voltage in the capacitor, which is proportional to the charge accumulated. In analogy to the membrane potential of the soma of a biological neuron, we denote this potential as V_MEM_ = Q/C. Where Q is the charge of the capacitor.

**Figure 2:**
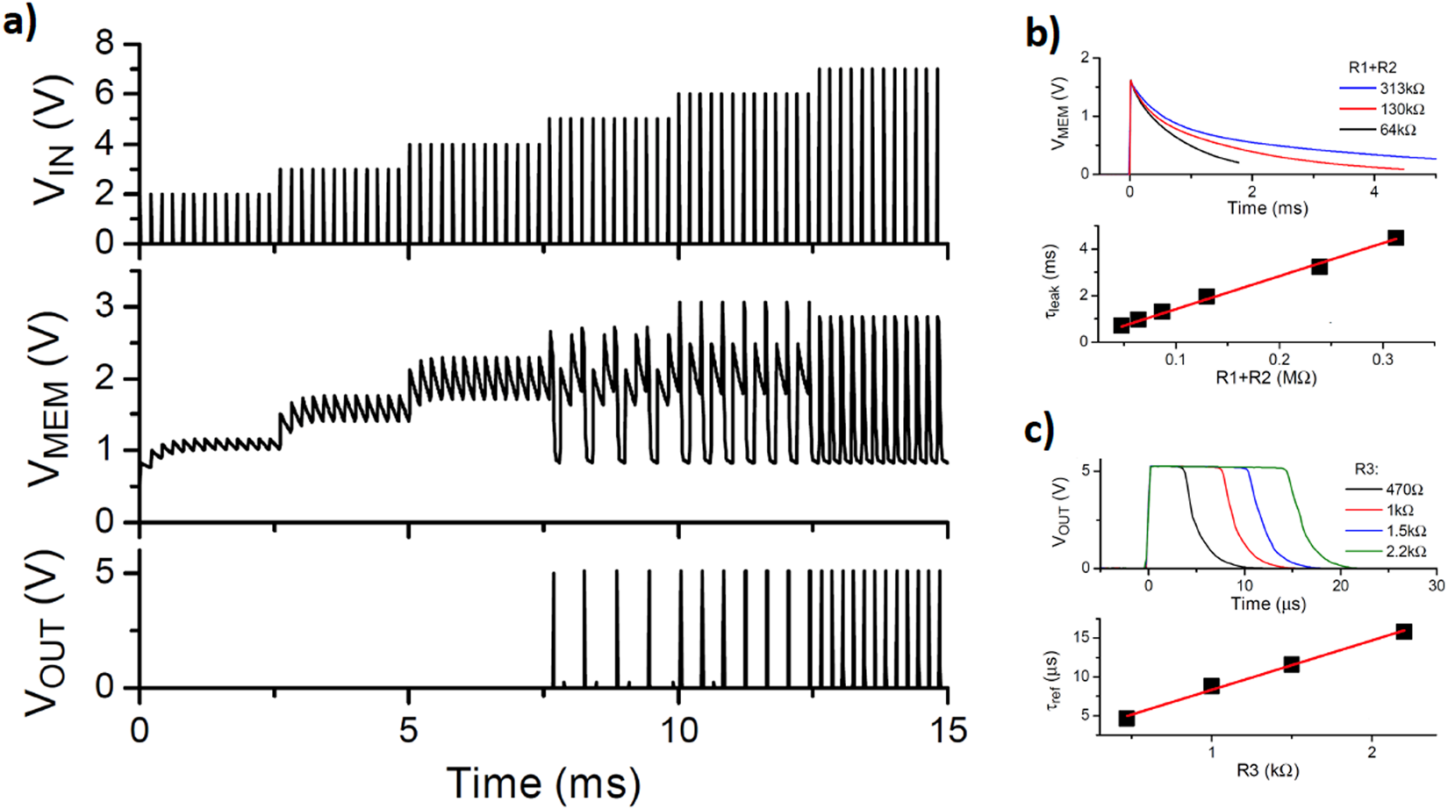
Basic behavior of the leaky-integrate-and-fire UC neuron. The signals shown on the left panels (a) are the measured voltages as a function of time for the input spikes, the membrane (capacitor V_C_) and the generated output spikes. The presence of a threshold for the incoming excitation is clearly observed. The right panels illustrate the detail line-shape and characteristic times of the UC neuron circuit. The upper panels (b) show the discharge (leak) of the capacitor, and the lower panels (c) the output spike signal generation.

In Fig. 2 we show the LIF behavior of the basic neuron block circuit introduced in the previous section. We apply as input a succession of voltage pulses of 10 μsec duration at 100 μsec interval and with increasing amplitude from 2 to 7 V. We observe the integrate and leaky features of the charge, which is reflected in the behavior of V_MEM_(t). When the input-spike voltage attains 5 V (this value also depends on the input-spike frequency) we observe a qualitative change in the behavior of the neuron, as its output begins to generate voltage spikes. This corresponds to the SCR switching to the on-state and allowing the capacitor to quickly discharge through it. We observe, also in agreement with the LIF model^8^, that as the incoming input spikes become more intense, the frequency of the outgoing spikes increases. This feature corresponds to the so called frequency or rate coding of neurons^10^. To demonstrate the ease of control and tunability of the UC neuron circuit, we have explored the dependence of the characteristic times with the resistive parameters. For the leaky time τ_leak_, we obtained the anticipated behavior, with τ_leak_ ~ (R_1_ + R_2_)C, as seen in Fig. 2 (panels b). We also considered the “refractory” time τ_ref_, which corresponds to the characteristic time of the generation of an outgoing spike when the SCR switches and remains in the on-state. The time τ_ref_ is approximately set by the discharge of C through the resistor R_3_, ie, τ_ref_ ~ R_3_C as seen in Fig. 2 (panels c).

An important requirement for a neuron circuit is the ability to drive downstream neurons with the generated output spike. The strength of the signal that comes out from the SCR, however, is limited by the stored charge and is insufficient for such a goal. Thus, we need to strengthen the output. A simple solution for this can be implemented by feeding the signal at the cathode of the SCR V_R3_ into a couple of MOS transistors T_1_ and T_2_ (which may be implemented with a CMOS pair in an integrated circuit). This portion of the circuit (blue box in Fig. 1) plays the role of the axon of the neuron.

We now demonstrate another basic and biologically relevant behavior of our UC neuron model, namely spike-frequency adaptation. This neuromorphic functionality can be achieved by adding a feedback loop.

**Figure 3:**
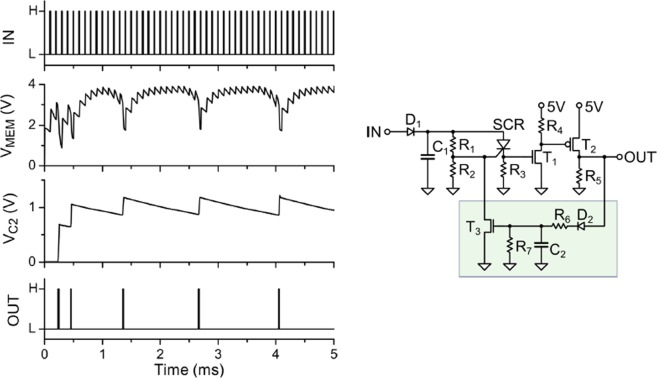
Spike-frequency adaptation of the leaky-integrate-and-fire UC neuron. The left panel shows, from top to bottom: Input voltage spikes, membrane voltage at C_1_, feedback loop voltage at C_2_, output spikes. We observe the decrease of the output spike-frequency in response to an input excitation with a constant spike-frequency rate. Right panel: Electronic circuit of the type II neuron, where a feedback loop (green box) is added to the type I neuron (*cf* Fig. 1).

The implementation is shown in Fig. 3, where the output signal is fed back to the gate of the SCR. This is done via the pair R_7_C_2_ that sets the characteristic time of the adaptation behavior, plus one additional transistor and a diode. The adaptive behavior is achieved by the variation of the trans-resistance of T_3_, which is in parallel with R_2_ at the gate of the SCR. The data in Fig. 3 show how the neuron that is subject to a constant incoming pulse-rate “adapts”, as its output spiking activity decreases from an initial high-rate to a lower one.

**Figure 4:**
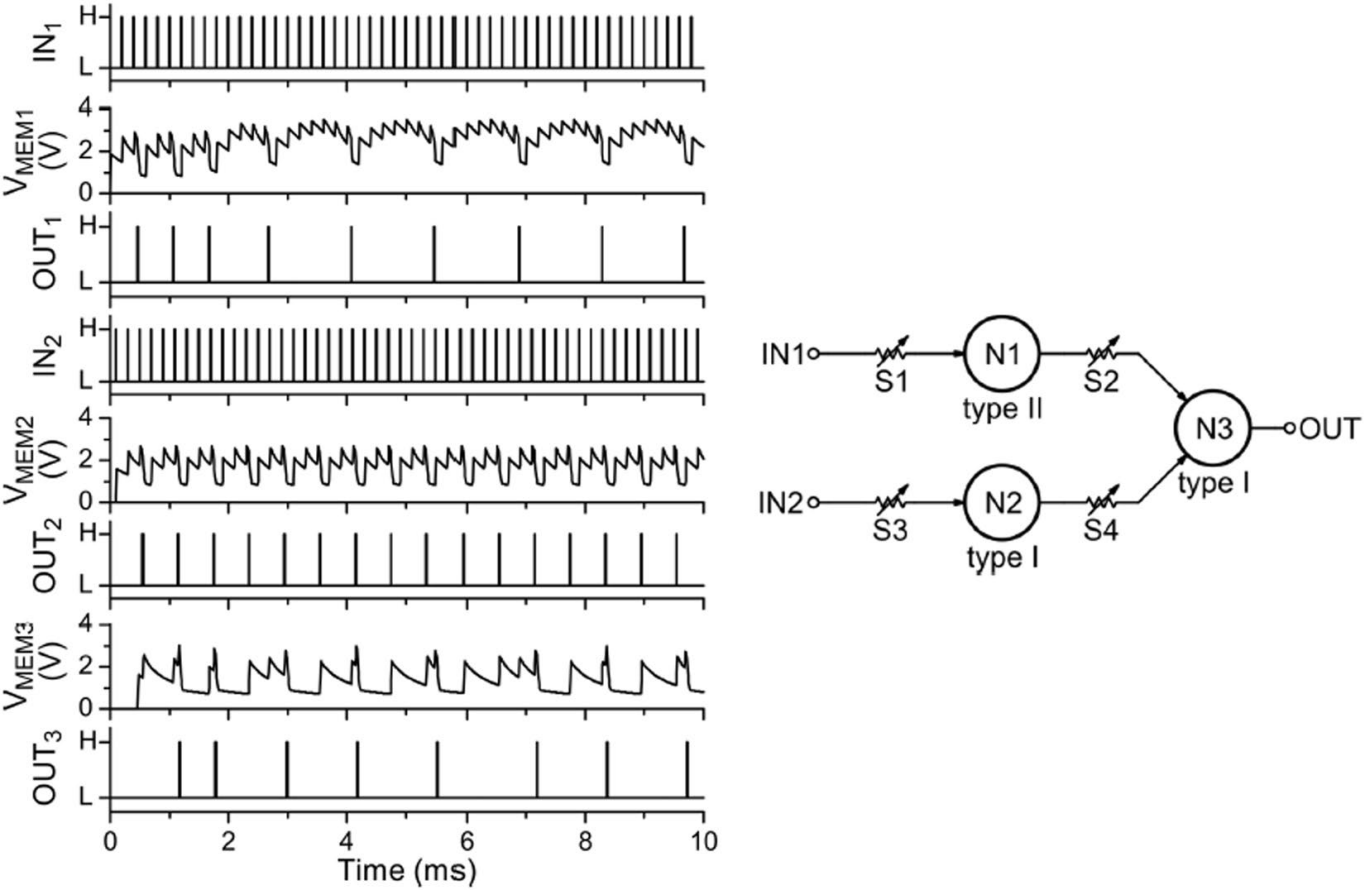
The right panel shows the schematic circuit that realizes a 2-layer spiking neuron network. The left panel shows the measured voltages as a function of time of the neurons N1, N2 and N3. N1 is a type II neuron with spike-frequency adaptation, N2 is a type I neuron and N3 is a type I neuron. The latter receives as input the addition of the outputs of N1 + N2, which results in a decreasing spiking rate at the output OUT_3_.

In Fig. 4 we describe one of the main results of our work. We demonstrate that our UC neuron is a module, ie, it is a building block for the straightforward construction of spiking neuron networks. Thus, multiple blocks can be interconnected as we illustrate with the elementary artificial neural network of three neurons forming a feedforward cascade. The circuit is depicted in the right panel of Fig. 4, where neurons N1 (type II) and N2 (type I) form the first layer and the neuron N3 (type I) forms the second layer. For simplicity, the synapses are 10 KΩ variable resistors. In general, these resistors may be replaced by memristors, which may also have a diode in series to avoid the sneak-path problem in large cross-bar arrays^11^. A key feature of this multi-layer neural network is that the post-synaptic neuron N3 is driven by the sum of the non-synchronous outputs of the pre-synaptic neurons N1 + N2. The fact that N3 actually responds to the sum of N1 + N2 is made evident by our choice of N1 as a type II neuron with spike-frequency adaptation. Inputs IN_1_ and IN_2_ to the network have constant spike rates, they produce different excitation of N1 and N2 (OUT_1_ and OUT_2_, respectively). These outputs are combined with equal (synaptic) weights as input to N3. This second layer neuron therefore receives an excitation with an overall decreasing rate, which results in a spiking activity (V_MEM3_ and OUT_3_) with a decreasing rate as well.

## Discussion

As can be seen from the data of Figs 2, 3 and 4, the typical firing time-scale is in the range of ms, which is comparable with that of biological neurons. This feature may enable implementing models of animal perception or navigation^12^ that could run in real time on a robot. On the other hand, more elaborate compact neuron implementations, such as, for instance Spikey^13^ run on much faster time scales. Those may be better adapted for more demanding computational capabilities, such as pattern recognition. In any case, the speed of the UC neuron is essentially settled by the RC time constant. With R in the 100 kΩ range and C in the 10nf one (see Table 1), we get RC ~ ms. Nevertheless, decreasing C to the pf range may increase the speed of the circuit by orders of magnitude, and this will not be limited by either the SCR or the transistors, which have relatively fast response times.

Regarding the relevant question of power consumption, an interesting feature of our UC circuit is that it is “normally off”. This makes it a priori power efficient, since the currents are negligible unless during the spike generation. While the question of global power dissipation of a network is not a simple matter, we may try to make some estimates for our circuit. Given a single neuron block, we may consider two different limiting cases: When the input-pulse frequency is high with respect to the 1/τ_leak_, and when it is much lower. In the former case the capacitor integrates the incoming pulses until the voltage V_C1_ reaches the fire threshold. Then, as leakage losses can be neglected, the energy dissipated per spike is E ~ C_1_V_pulse_^2^/2. This is the energy stored in C_1_. Taking V_pulse_ of the order of a volt, then one may expect E ~ 1pJ for a neuron implemented in an integrated circuit. In the second limiting case the input pulses are separated, then if N pulses are necessary to excite one output spike, an upper bound for the energy per spike would be E ~ N[C_1_V_pulse_^2^/2]. However, the *power* in this latter case would be lower than in the former one, because the time between output spikes (~Nτ_leak_) would be relatively much longer.

We may put the previous discussion in a broader context. The power consumption of a spiking neural network depends on the energy per spike of neurons and also on their spike rate. A rough estimate for spike rate of neurons in the human brain cortex is 1–10 Hz. Hence, considering 10^11^ the number of neurons and the energy of 1pJ that we estimated for the UC neuron, we get (10/s 10^11^ 10^−12^ J) ~ 1 W, which gives the order of magnitude of the human brain cortex. However, the large size of capacitors remains a limiting factor for an integration of 10^11^ units. Alternatively, we may estimate the spiking rate corresponding to energy per spike evaluated above E ~ 1pJ as the inverse time-constant 1/RC ~ 1/(100 kΩ 1pf) ~ 0.1/μs. Thus, for a power consumption of 1 W we get [1 W/(0.1/μs 1pJ)] ~ 10^7^ neurons, an order of magnitude larger than the number of neurons of a TrueNorth chip. While these estimates are rough lower bounds since they do not include the consumption of the synapses, they indicate that a spiking neuron network based on UC units may be competitive and has still room to improve.

Another aspect to consider in regard to neural networks implementation is related to the learning or training capability. In practice, this may be done either off-line, by simulations to determine the parameters of the network; or on-line, via an automatic feed-back loop. The actual implementation would depend on the desired functionality of the network and is a vast topic that is outside the scope of the present work. Nevertheless, we may discuss some general considerations relevant to our present UC neuron. In the case of spiking neural networks, the parameters may be the synaptic weights, which are resistors that interconnect the neurons, such as depicted by the resistors S_i_ indicated in Fig. 4, or it may also be the neuron *internal* parameters. For instance, relaxation time, integration time, threshold voltage, adaptation time, etc., can be adjusted by direct tuning of the UC neuron resistor values. An appealing feature of our circuit is that its simplicity allows for a rather straightforward control of these variables as shown in the data of Fig. 2. Tunable resistors with memory or memristors^2^ are very well adapted for these tasks. In the case of Fig. 3 we demonstrated how a simple feed-back loop at the gate of the SCR allows for the control of the firing rate of the neuron.

The UC neuron circuit is built around an SCR whose key feature is a non-linear I-V characteristic with a voltage threshold for conduction. This threshold can be controlled by the *gate* voltage, which was crucial for implementing the spike-frequency adaptation. In addition, the SCR displays *hysteresis* behavior, since the conduction state is switched off when the current is beneath a low hold-current threshold. This feature permits the control of the spike duration and the refractory time.

Besides the already mentioned challenge for VLSI to reduce the footprint of the membrane capacitor, to implement the UC neuron crucially depends on the possibility of realizing the SCR (or the non-linear SCR characteristics) with a VSLI compatible technology. This issue is beyond the scope of the present work and our UC neuron circuit is at the proof-of-concept level. In any case, there are no *a priori* impediments to integrate the *pnpn*-junction structure of the SCR device and implementations were already reported in the literature^9^. While this appears to be an open road to pursue, one should also bear in mind that there are other possibilities. In fact, as we already briefly mentioned before, Mott materials may also be taken into consideration. These, so called, strongly correlated insulators, such as VO_2_, V_2_O_3_, NdNiO_3_, etc., display qualitatively similar I-V characteristics to that of the SCRs. The key physical phenomenon in those systems is an unusual thermally driven first-order insulator-metal transition, which may also be induced by a strong electric field^14–16^. An important and attractive feature is that while the Mott materials are challenging to control and fabricate, they may eventually enable the replacement of the whole SCR + RC block of the “soma” with a single two-terminal Mott insulator device^17,18^. This would provide further simplicity and power efficiency for the implementation of the ultimate ultra-compact neuron^2^.

## Methods

The neuron circuits in this work were all implemented with out-of-the-shelf components that we list below.

**Table 1:** For the input and measured voltages, we used six analog input and two analog outputs of a National Instrument multichannel acquisition system (NI PXIe-6289), respectively.

D (Fig. 1), D1 (Fig. 3), D2	1N4148
C (Fig. 1), C1 (Fig. 3)	15 nF
R1	68 kΩ
R2	390 kΩ
SCR	CR02A
R3	1.5 kΩ
T1, T3	IRLML6246TR
R4	10 kΩ
T2	ZVP3310A
R5, R6	4.7 kΩ
C2	22 nF
R7	100 kΩ

The analog inputs used to measure the capacitor voltages where buffered using a Texas Instrument operational amplifier (TL-084). We developed an ad-hoc acquisition software based on LabVIEW. The generation of the input voltages was at a rate of 100ksps. The acquisition of the input voltages was done synchronously. The oscillograms in Fig. 2 were acquired using an Agilent digital oscilloscope (DSO6104A) with standard (non-attenuated) probes.

## Conclusions

In this work we have introduced an ultra-compact circuit for a LIF artificial neuron, which realizes a basic building block for constructing spiking neural networks. The key characteristic times can be easily tuned by resistive parameters. It is based on an SCR and is implemented with very few conventional out-of-the-shelf electronic components. Their number is likely minimal, as we have identified each one of the three features of the leaky, integrate and fire model with three components, a resistor, a capacitor, and a SCR, respectively. We demonstrated that the UC circuit has the following features: *(i)* the output of a (pre-synaptic) neuron can trigger a downstream (post-synaptic) one; *(ii)* the addition of a feedback line implements spike-frequency adaptation; *(iii)* the UC block modules can be interconnected to build multi-layer neuron network structure. Furthermore, our UC circuit has low power consumption, as it is always in the off-state, unless during the brief spike generation. The dissipated power was argued to be mainly due to the discharge of the capacitor. Thus, upon integration one may expect to reach an energy consumption of a pJ per spike or less. The simplicity of our ultra-compact neuron opens an exciting way to achieve the large-scale multi-layer neural networks that are required for the ongoing quest to mimic the human brain.

## References

[1] Sejnowski, T. J. *The Deep Learning Revolution*. (The MIT Press, 2018).

[2] Del Valle J, Ramirez JG, Rozenberg MJ, Schuller IK (2018). Challenges in materials and devices for resistive-switching-based neuromorphic computing. J. Applied Phys..

[3] Silver D (2016). Mastering the game of Go with deep neural networks and tree search. Nature.

[4] Merolla PA (2014). Artificial brains. A million spiking-neuron integrated circuit with a scalable communication network and interface. Science.

[5] Thakur CS (2018). Large-Scale Neuromorphic Spiking Array Processors: A Quest to Mimic the Brain. Front Neurosci.

[6] Chicca E, Stefanini F, Bartolozzi C, Indiveri G (2014). Neuromorphic Electronic Circuits for Building Autonomous Cognitive Systems. Proc. IEEE.

[7] Indiveri G (2011). Neuromorphic Silicon Neuron Circuits. Front Neurosci.

[8] Gerstner, D. W., Kistler, W. M., Naud, R. & Paninski, L. *Neuronal Dynamics: From Single Neurons to Networks and Models of Cognition* (Cambridge University Press, 2014).

[9] Ker M-D, Hsu K-C (2005). SCR device fabricated with dummy-gate structure to improve turn-on speed for effective ESD protection in CMOS technology. IEEE Trans. Semicond. Manuf..

[10] Adrian ED, Zotterman Y (1926). The impulses produced by sensory nerve endings: Part II: The response of a single end organ. J Physiol..

[11] Xu, C. *et al*. Overcoming the challenges of crossbar resistive memory architectures. *2015 IEEE 21st International Symposium on High Performance Computer Architecture (HPCA)*, Burlingame, CA, pp. 476–488 (2015).

[12] Jeffress LA (1948). A place theory of sound localization. J Comp Physiol Psychol.

[13] Pfeil T (2013). Six networks on a universal neuromorphic computing substrate. Front. Neurosci..

[14] Zimmers A (2013). Role of Thermal Heating on the Voltage Induced Insulator-Metal Transition in VO_2_. Phys. Rev. Lett..

[15] Parihar A, Jerry M, Datta S, Raychowdhury A (2018). Stochastic IMT (Insulator-Metal-Transition) Neurons: An Interplay of Thermal and Threshold Noise at Bifurcation. Front. Neurosci..

[16] Stoliar P (2013). Universal Electric-Field-Driven Resistive Transition in Narrow-Gap Mott Insulators. Adv. Mater..

[17] Stoliar P (2017). A Leaky-Integrate-and-Fire Neuron Analog Realized with a Mott Insulator. Adv. Funct. Mater..

[18] Tesler F (2018). Relaxation of a Spiking Mott Artificial Neuron. Phys. Rev. Applied.

